# Aflatoxin Exposure During Pregnancy, Maternal Anemia, and Adverse Birth Outcomes

**DOI:** 10.4269/ajtmh.16-0730

**Published:** 2017-04-05

**Authors:** Laura E. Smith, Andrew J. Prendergast, Paul C. Turner, Jean H. Humphrey, Rebecca J. Stoltzfus

**Affiliations:** 1 Division of Nutritional Sciences, Cornell University, Ithaca, New York; 2 Zvitambo Institute for Maternal and Child Health Research, Harare, Zimbabwe; 3 Blizard Institute, Queen Mary University of London, London, United Kingdom; 4 Department of International Health, Johns Hopkins Bloomberg School of Public Health, Baltimore, Maryland; 5 Maryland Institute for Applied Environmental Health, School of Public Health, University of Maryland, College Park, Maryland

## Abstract

Pregnant women and their developing fetuses are vulnerable to multiple environmental insults, including exposure to aflatoxin, a mycotoxin that may contaminate as much as 25% of the world food supply. We reviewed and integrated findings from studies of aflatoxin exposure during pregnancy and evaluated potential links to adverse pregnancy outcomes. We identified 27 studies (10 human cross-sectional studies and 17 animal studies) assessing the relationship between aflatoxin exposure and adverse birth outcomes or anemia. Findings suggest that aflatoxin exposure during pregnancy may impair fetal growth. Only one human study investigated aflatoxin exposure and prematurity, and no studies investigated its relationship with pregnancy loss, but animal studies suggest aflatoxin exposure may increase risk for prematurity and pregnancy loss. The fetus could be affected by maternal aflatoxin exposure through direct toxicity as well as indirect toxicity, via maternal systemic inflammation, impaired placental growth, or elevation of placental cytokines. The cytotoxic and systemic effects of aflatoxin could plausibly mediate maternal anemia, intrauterine growth restriction, fetal loss, and preterm birth. Given the widespread exposure to this toxin in developing countries, longitudinal studies in pregnant women are needed to provide stronger evidence for the role of aflatoxin in adverse pregnancy outcomes, and to explore biological mechanisms. Potential pathways for intervention to reduce aflatoxin exposure are urgently needed, and this might reduce the global burden of stillbirth, preterm birth, and low birthweight.

## Introduction

Aflatoxins are toxic secondary metabolites of *Aspergillus* molds that contaminate foods such as maize, rice, and legumes. Around 0.5 billion people, predominantly those living in developing countries, are at significant risk of exposure to dietary aflatoxins, with many people chronically exposed to aflatoxins throughout life.^1^ In many developing countries, aflatoxins are not effectively controlled in the food system and consumption of high-risk foods, such as maize and groundnuts, is common. Aflatoxin accumulation in food is highly dependent on environmental factors such as moisture, temperature, nitrogen availability, and plant density,^2^ as well as poor harvest practices and improper grain storage.^3^–^6^ Aflatoxins have been most widely studied as causative agents of liver cancer.^7^ Chronic exposure has also been associated with other adverse human health outcomes, including growth faltering^8^ and maternal anemia.^9^ Exposure during pregnancy has been widely documented,^10^,^11^ but the effects on the mother and fetus are not well described.

Aflatoxins inhibit protein synthesis, are cytotoxic, teratogenic, and immunotoxic.^12^,^13^ Thus, they may affect fetal health both directly during critical periods of development, or indirectly, through their adverse effects on maternal health. Accordingly, aflatoxin exposure during pregnancy may plausibly contribute to adverse pregnancy outcomes, including intrauterine growth restriction, premature delivery, and pregnancy loss.^14^ If this is the case, the public health implications could be substantial: globally there are 2.65 million stillbirths,^15^ 32 million small-for-gestational-age,^16^ and 15 million premature deliveries^17^ every year.

Adverse birth outcomes stem from multiple causes, many of which remain poorly understood. Advanced maternal age, parity, maternal infection, and smoking are well-documented risk factors for adverse birth outcomes, but do not account for a large proportion of cases.^18^ Maternal anemia during the first and second trimesters has also been associated with preterm birth and low birthweight, although the causal mechanism is not well understood.^19^–^21^ Maternal inflammation is the only pathologic process with a clearly defined causal link to preterm birth.^22^ Notably, aflatoxin exposure in humans and animals has been associated with increases in inflammatory markers,^23^–^25^ suggesting a potential mechanism pathway linking aflatoxin exposure and adverse birth outcomes. Anemia of inflammation might also occur, and could mediate prematurity or poor fetal growth.

Recently, documented high prevalence of exposure during pregnancy has directed focus on aflatoxins as a potential harmful exposure during the first 1,000 days^23^,^26^—the developmentally sensitive period from conception to 2 years of age. There are no published randomized controlled trials evaluating the effect of aflatoxin exposure during pregnancy. Therefore, we aimed to conduct a narrative review to evaluate the evidence for a potential role of aflatoxin exposure and anemia in intrauterine growth retardation, preterm birth, and pregnancy loss.

## Methods

We searched ISI Web of Knowledge and PubMed (with medical subject headings [MeSH] strings) using these search terms: preterm, low birthweight, fetal loss, fetal resorption, hemolysis, anemia, iron deficiency, and miscarriage, with mycotoxin or aflatoxin in the search, between the years 1970 and 2015. We identified additional relevant papers from related review articles identified in the primary search. A total of 27 studies (10 humans, 17 animals) assessing the relationship between aflatoxin exposure and adverse birth outcomes or anemia were identified. A second search was conducted to identify literature evaluating mechanisms that could mediate these adverse health outcomes, using these terms: placenta, cytokines, interleukin-1, interleukin-6 (IL-6), tumor necrosis factor-α, insulin-like growth factor, intestine, inflammation, immune, and organ, with mycotoxin or aflatoxin in the search.

### Aflatoxin metabolism and implications for exposure during pregnancy.

Aflatoxins B1 (AFB1), B2, G1, and G2 are produced by several species of *Aspergillus*; AFB1 exposure is the focus of most research because it occurs most frequently and is most toxic.^27^ All aflatoxins are readily absorbed and undergo a variety of biotransformation reactions; both the parent aflatoxins and their metabolites are detectable in urine. AFB1 becomes toxic through metabolic activation by various cytochrome P450 enzyme families including CYP1a2, CYP3a4, and CyP3a5, which are mainly found in the liver, but also present in other tissues including the placenta, intestine, and spleen.^12^ Activation generates two reactive epoxide species, AFB1-8,9-exo-epoxide and AFB1-8,9-endo-epoxide, and several other metabolites including aflatoxin M1 (AFM1). The aflatoxin epoxides and AFM1 are toxic; AFB1 exo-epoxide binds to DNA forming mutagenic lesions.^13^

The epoxides can be detoxified by glutathione-S transferases (GST) before urinary excretion as aflatoxin mercapturates.^28^ They may also be enzymatically hydrolyzed, and then detoxified to a dialchohol.^29^,^30^ Enzymes involved in both activation and detoxification of aflatoxin are polymorphic, and can therefore influence the toxic insult of exposure.^31^ GST polymorphisms are relatively common and have been associated with risk of various cancers as well as adverse birth outcomes.^32^–^34^

In evaluating the available evidence for a potential role of aflatoxin exposure in adverse pregnancy outcomes, it is important to recognize variations in the balance of activation versus detoxification depending on dose, species, and age. A fetal form of CYP3a4, known as CYP3a7, has been observed in fetal liver within 2 months of conception,^35^,^36^ indicating that the fetus may metabolically generate reactive epoxides following transplacental transfer of maternally ingested AFB1.^37^ Fetal livers catalyze the formation of the epoxide at similar rates to adults but produce fewer GSTs, and thus have a lower capacity to protect against toxicity.^38^ In addition to age differences and genetic differences in aflatoxin metabolism, there is also considerable interspecies variability in aflatoxin metabolism. A review by Wild and others^39^ suggested that humans may be relatively sensitive to the effects of aflatoxin, as higher levels of aflatoxin-albumin (AF-alb) were formed per dose of AFB1 in humans compared with many standard laboratory animals.

### Human biomarker measurement.

The human studies identified in this review relied on biomarkers to assess aflatoxin exposure; no trials involving experimental manipulations of the diet during pregnancy were identified. Dietary aflatoxins and their metabolites can be detected in blood, urine, and breast milk, but their concentrations are not equally associated with dietary intake. There are three validated biomarkers of aflatoxin exposure: urinary biomarkers reflecting exposure in the prior 24–48 hours (AFM1 and aflatoxin-N7-guanine) and serum biomarkers reflecting cumulative exposure over the prior 2–3 months (AF-alb). These biomarkers are all quantitatively associated with dietary aflatoxin intake.^40^ Other aflatoxin metabolites (e.g., serum AFM1 or AFG1, urinary AFB1 or AFG1, and milk AFM1 or AFG2) are indicative of exposure, but levels do not correlate with dietary intake and they are therefore termed biomeasures. This difference is important when comparing the strength of epidemiological data that describe relationships between aflatoxin and health outcomes.

### Prevalence of aflatoxin exposure during pregnancy.

We identified 12 epidemiologic studies from Africa, Asia, and the Middle East, including a total of more than 2,000 participants, which reported AF exposure in pregnant women and/or infant cord blood. Of these studies, eight measured AF exposure in both cord blood and maternal blood, whereas four measured exposure only in cord blood. Prevalence of exposure ranged from 6% to 100%,^23^,^41^–^51^ suggesting that in utero exposure to aflatoxin is widespread where maternal diets are contaminated. Cord blood samples in Taiwan were found to have AF-DNA adducts,^49^ confirming both fetal aflatoxin exposure and biotransformation capacity to generate reactive aflatoxin-epoxides.

### Aflatoxin and intrauterine growth restriction.

Although exposure is widely documented, there are few human studies examining the relationship between AF exposure and pregnancy outcomes. We found four human studies that relied on aflatoxin biomeasures; three reported a negative association between an aflatoxin biomeasure and birthweight,^46^,^47^,^51^,^52^ and one reported no association^42^ (Supplemental Table 1). The presence of AFB1 and AFM1 in cord blood is quite transient, and in the Nigerian study by Maxwell and others,^42^ only 14.6% of samples were positive for aflatoxins. These studies may have inconsistent findings in part because the indicators of aflatoxin exposure used were biomeasures, which represent transient exposure and are not correlated with dietary intake. We identified two studies that used quantitative biomarkers, and both of these reported significant associations. A study of 785 pregnant Ghanaian women found that those in the highest quartile of AF-alb had significantly greater odds of having a low birthweight infant compared with the lowest quartile, with a linear trend of increasing risk of low birthweight with ascending aflatoxin quartile.^14^ In a study of 119 Gambian mother-infant pairs, average maternal AF-alb (at 5 and 8 months gestation) was significantly associated with lower weight-for-age (−0.249 *z* scores, *P* = 0.012) and lower height-for-age (−0.207 *z* scores, *P* = 0.044).^48^

We identified nine experimental animal studies, which reported consistent significant adverse effects of aflatoxin on fetal growth. Animal studies in rats, mice, hamsters, swine, rabbits, and quail consistently report decreased fetal weight, crown-rump length, and organ weight among exposed animals compared with control animals across a wide range of aflatoxin doses (Supplemental Table 2).

### Aflatoxin, fetal loss, and spontaneous preterm birth.

We did not identify any human studies investigating risk of fetal loss, and only one study investigating preterm birth in association with aflatoxin exposure during pregnancy. We only found two studies investigating aflatoxin and stillbirth; both reported high levels of aflatoxin biomeasures in cord blood of three stillborn infants, though neither study was designed to assess a causal relationship nor investigate a mechanism^41^,^51^ (summarized in Supplemental Table 1). A study in Ghana in which gestational age was measured by ultrasound or palpation during routine antenatal care found no relationship between preterm birth (< 37 weeks gestation) and AF-alb biomarkers.^14^ Although these methods are relatively accurate for determining gestational age in the first 20 weeks, in Ghana, 43% of women have their first antenatal care (ANC) visit after the first trimester.^53^ In addition, the best methods for gestational age dating include an error of ±week that can lead to misclassification of prematurity.^54^,^55^

In animals, aflatoxin exposure during pregnancy causes fetal anomalies and decreased live births and litter size. In a study assessing aflatoxin exposure in pregnant rabbits, animals were dosed with 0–100 μg AF/kg body weight (bw)/day.^56^ Wangikar and others^56^ reported a decreased percentage of live fetuses and increased resorption (∼5%), impaired organ development (14% reduction in organ weight in 100 μg AF/kg bw/day treatment), and skeletal anomalies (28% of offspring with anomalies in 100 μg AF/kg bw/day). Similarly, Kihara and others^57^ found that rats treated with aflatoxin (300 μg AF/kg bw/day) on day 15–18 of gestation had a lower proportion of live births/implants (85%) compared with controls (94%). Aflatoxin is a potent teratogen because of its ability to bind DNA and subsequently inhibit protein synthesis. Several studies have reported skeletal anomalies in the offspring of animals treated with aflatoxin during pregnancy.^56^,^58^–^60^ The doses of aflatoxin used in these animal experiments are similar to estimated exposures ranging from 0 to 91 μg AF/kg bw/day in human studies in Zimbabwe and China.^61^

### Aflatoxin and maternal anemia.

Aflatoxin exposure has been associated with anemia in one human study and several animal studies. Shuaib and others^9^ reported a cross-sectional association between AF-alb and anemia in 785 pregnant Ghanaian women, with those in the highest quartile of aflatoxin exposure having 1.85-fold increased odds of anemia compared with those in the lowest quartile (confidence interval = 1.16–2.85).

In vitro and in vivo studies in dogs, rabbits, catfish, and poultry show that relatively high aflatoxin exposures (range 0.5–1 ppm) are cytotoxic and cause lysis of red blood cells.^62^–^65^ This small body of evidence (summarized in Supplemental Table 3) indicates that aflatoxin may cause low hematocrit and hemoglobin,^66^,^67^ low iron absorption,^68^ and microcytic hypochromic anemia,^69^ all of which are characteristic of iron deficiency.

**Figure 1.**
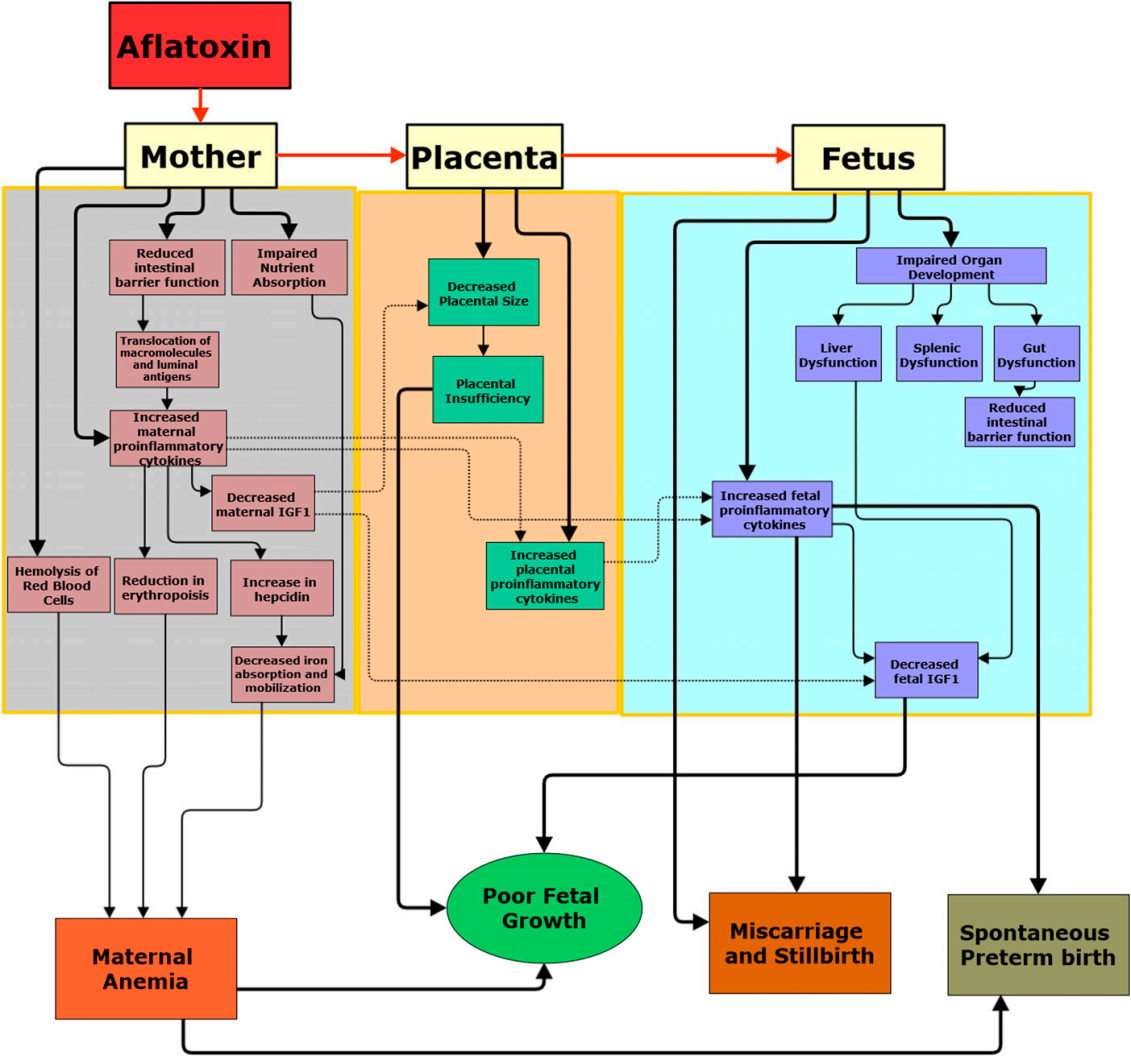
(**A**) Conceptual framework for the effect of aflatoxin exposure on maternal-fetal health. Maternal exposure to aflatoxin might cause adverse pregnancy outcomes through three primary pathways: induction of environmental enteric dysfunction characterized by intestinal inflammation, impaired barrier function, and systemic immune activation; upregulation of pro-inflammatory cytokines and downregulation of anti-inflammatory cytokines; and potential toxic effects on maternal and fetal organs once absorbed causing systemic immune activation, and impaired placental and fetal development. Red arrows represent the transfer of aflatoxin from mother to placenta to fetus. Solid black arrows represent the hypothesized effects of aflatoxin exposure in the mother, placenta and fetus; dotted black arrows represent the hypothesized indirect effect of aflatoxin-induced maternal inflammation on the placenta and fetus. The few cross-sectional studies conducted in humans suggest that aflatoxin exposure impairs intrauterine growth and may be a cause of pregnancy loss. Although no conclusive studies have been conducted in humans, we have reviewed evidence from in vivo and in vitro studies that aflatoxin exposure may cause anemia, intestinal damage, and elevation of pro-inflammatory cytokines. In addition, animal studies indicate aflatoxin exposure results in impaired organ development. There are no studies investigating the effects of aflatoxin on the placenta, but given that the placenta metabolizes and transports aflatoxin, further investigations into its effects are warranted. This framework provides directions for future research investigating the effects of aflatoxin exposure during pregnancy.

Although in vivo and in vitro studies have focused on a red cell lysis pathway, doses to cause such an effect are likely higher than levels experienced in human populations. It is more probable that chronic aflatoxin exposure could therefore cause anemia through three different mechanisms related to immune activation and enteropathy: a decreased capacity of the intestine to absorb essential nutrients such as iron; a decrease in erythropoiesis arising from chronic inflammation; and reduced availability of iron due to hepcidin upregulation (Figure 1

). IL-6—which has shown to be upregulated by aflatoxin exposure^24^—increases hepcidin production, which decreases iron absorption and iron release from macrophages.^70^ However, further human and animal studies are needed to test these hypotheses. In summary, although it is biologically plausible that aflatoxin exposure causes anemia, the doses used in animal studies are high and the results may not be relevant for human exposure. Although there is evidence that aflatoxin exposure can alter the inflammatory response, none of the studies reviewed explored aflatoxin exposure and inflammation-induced anemia.

### Interactions between aflatoxin exposure and maternal diet.

Pregnant women in developing countries have multiple overlapping risk factors for poor pregnancy outcomes, which may increase vulnerability to even low doses of aflatoxin. For example, when diets are deficient in vitamins A, C, or E, or selenium (all of which protect against the toxic effects of aflatoxin), the detoxifying system for aflatoxin may be impaired, increasing the production of epoxides.^71^,^72^ Populations at risk of frequent aflatoxin exposure are often those with poor dietary diversity and thus micronutrient insufficient is common. We did not identify animal studies designed to explore potential interactions between nutritional deficiencies and aflatoxin exposure.

### Potential mechanisms linking aflatoxin exposure to adverse pregnancy outcomes.

Mechanistic studies suggest that maternal exposure to aflatoxin might cause adverse pregnancy outcomes through four primary pathways: 1) upregulation of pro-inflammatory cytokines and/or downregulation of anti-inflammatory cytokines^23^,^24^,^73^–^76^; 2) induction of enteropathy characterized by intestinal inflammation and impaired barrier function, leading to systemic immune activation^24^,^76^–^78^; 3) potential toxic effects on maternal organs causing systemic immune activation and impaired placental and fetal development^24^,^70^,^79^,^80^; and 4) toxic effects on fetal organs causing fetal inflammation and impaired fetal development^56^,^81^–^83^ (Figure 1).

This framework offers numerous hypotheses suggested by the current literature, with proposed mechanisms drawing heavily on evidence from in vivo animal studies and in vitro studies. We highlight two necessary areas for future research to refine and test these hypotheses. First, there is a need for rigorous human studies on the relationship between maternal aflatoxin exposure and adverse pregnancy outcomes, using validated biomarkers. Although cross-sectional studies at delivery would be informative, stronger designs would investigate the longitudinal relationship of maternal exposure during pregnancy and pregnancy outcomes or experimentally intervene to reduce aflatoxin exposure in maternal diets. Second, research is needed to elucidate the mechanism by which aflatoxin mediates adverse pregnancy outcomes. For translational science in particular, studies are needed to understand the effects of aflatoxin on the placenta and fetus at modest dose and on a background of maternal dietary insufficiency, as this commonly co-occurs with aflatoxin exposure in human populations.

## Conclusion

Aflatoxin exposure is common in developing countries, making it an issue of substantial public health importance. Despite this, there are relatively few human studies investigating the effects of aflatoxin during pregnancy on the mother and fetus. Aflatoxin exposure assessment studies have been small and geographically scattered and the majority of human studies have focused on low birthweight. However, animal studies provide biological support for the hypothesis that aflatoxin exposure may mediate adverse pregnancy outcomes.

Given the enormous burden of aflatoxin exposure, anemia, intrauterine growth restriction, and preterm birth in developing countries in the context of co-exposure to multiple environmental insults, further investigations into the effects of aflatoxin are strongly warranted. Research is needed to comprehensively evaluate the relationship and potential mechanisms linking aflatoxin with adverse health outcomes in pregnancy, so that novel pathways for intervention can be defined to mitigate aflatoxin exposure and reduce the global burden of stillbirth, preterm birth, and low birthweight.

## Supplementary Material

Supplemental Tables.
