# Supplementary material for: Aflatoxin Exposure During Pregnancy, Maternal Anemia, and Adverse Birth Outcomes
Source: Am J Trop Med Hyg. 2017 Apr 5;96(4):770–6. doi: 10.4269/ajtmh.16-0730 (PMC5392618; doi:10.4269/ajtmh.16-0730)
Supplement: Supplementary file 1 [file SD1.pdf]

Supplemental Table 1

Human studies on the effect of aflatoxin exposure during pregnancy

Author (year)	Research questions (study design)	Population (country)	Biomarker or Biomeasure*	Number positive (%) in maternal or cord blood	Mean (range of exposure)	Outcome
De Vries and others (1989)[1]	Is in utero aflatoxin exposure associated with low birthweight? (cross-sectional)	125 mother-infant pairs (Kenya)	AFB1, AFB2, AFM1, AFM2, AFG1, AFG2, aflatoxicol	Maternal: 110 (53) Cord: 37 (37)	Range Maternal: AFM1 and AFM2 (12–1,689 pg/mL); AFB1 (89–1,1574 pg/mL) Cord: AFM1 and AFM2 (17–656 pg/mL); AFB1 (86–6,819 pg/mL)	Female infants exposed to aflatoxins had birthweights that were 255 g lower than nonexposed female infants. There were no significant differences in male infants. Two stillbirths had high levels of aflatoxins
Maxwell and others (1994)[2]	Is in utero aflatoxin exposure associated with low birthweight? (cross-sectional)	625 infants (Nigeria)	AFB1, AFB2, AFM1, AFM2, AFG1, AFG2, aflatoxicol	Cord: 91 (14.6)	Range Cord: AFM1 (32–11,354 ng/L); AFM2 (14–3,644 ng/L); AFB1 (168–69,973 ng/L) AFB2 (15–144 ng/L)	No correlation between aflatoxin exposure and birthweight
Abdulrazzaq and others (2002)[3]	Are AFM1 concentrations in mothers associated with low birthweight? (cross-sectional)	201 cord blood samples at birth (United Arab Emirates)	AFM1, AFM2, AFB1	Cord: AFM1: 107 (53); AFM2: 31 (15); AFB1: 27 (13)	Median (range) Cord: AFM1 1,229 pg/mL (110–4060); AFM2 1,207 pg/mL (210–3,700); AFB1 1,822 pg/mL (228–15,225)	There was a strong negative correlation between AFM1 and birthweight ($r = -0.63$)
Abdulrazzaq and others (2004)[4]	Do AFM concentrations in mothers and newborns correlate and is the presence of AFM associated with morbidity? (cross-sectional)	166 pregnant women and paired cord blood samples (United Arab Emirates)	AFM1	Maternal: 113 (68) Cord: 111 (67)	Mean (range) Maternal: 1.04 (0.03–8.49 ng/mL) Cord: 1.88 (0.05–10.44 ng/mL)	There was a strong correlation ($r = 0.797$, $P < 0.0001$) between maternal and cord blood levels of aflatoxin. There was also a strong negative correlation between aflatoxin levels and birthweight ($r = 0.565$, $P < 0.001$) but there was no association between aflatoxin M1 concentration in maternal or cord blood and rates of jaundice or infection
Turner and others (2007)[5]	What is the effect of in utero aflatoxin exposure on infant growth in the first year of life (longitudinal)	138 singleton infants (Gambia)	AFB1-lys	Maternal: 119 (100) Cord: 48 (48.5)	Median (IQR) Maternal: 38.9 pg/mg albumin (23.3–64.1 Cord: 2.5 pg/mg albumin (2.5–7.9)	In infants aged 52 weeks: 1) averaged maternal AF-alb (at 5 and 8 months gestation) was significantly associated with lower weight for age (–0.249 SD, $P = 0.0123$ and 2) average maternal AF-alb significantly associated with lower HAZ (–0.207 SD, $P = 0.044$)

(continued)

Supplemental Table 1

Continued

Author (year)	Research questions (study design)	Population (country)	Biomarker or Biomeasure*	Number positive (%) in maternal or cord blood	Mean (range of exposure)	Outcome
Shuaib and others (2010)[6]	What is the relationship between birth outcomes and blood levels of AF-lysine adduct in pregnant women? (cross-sectional)	755 pregnant women (Ghana)	AFB1-lys	N/A	Mean (range) Maternal: 10.9 pg/mg albumin (0.44–268.73)	Pregnant women with AF levels in highest quartile were 2.09 (CI = 1.19–3.68) times as likely to have low birthweight infants; other adverse birth outcomes were not significant but trended toward an increased risk
Lamplugh and others (1988)[7]	Do aflatoxins cross the placenta? (cross-sectional)	188 cord blood samples (Ghana) 77 mother infant pairs with maternal and cord blood (Nigeria)	AFB1, AFB2, AFM1, AFM2, AFG1, AFG2, aflatoxicol	Ghana Cord: 63 (34) Nigeria Maternal: 16 (77) Cord: 9 (12)	Range Ghana Cord: AFM1 = (34–7,320 ng/L); AFM2 = (30–572 ng/L); AFB1 = (185–43,822 ng/L) AFB2 = (11–925ng/L) Nigeria: N/A	Exposure in cord blood indicates that aflatoxin exposure crosses the placenta; one stillbirth recorded with high aflatoxin levels
Shuaib and others (2010)[8]	What is the association between anemia and aflatoxin exposure in pregnant women? (cross-sectional)	755 pregnant women (Ghana)	AFB1-lys	N/A	Mean (range) Maternal: 10.9 pg/mg albumin (0.44–268.73)	Aflatoxin exposure was significantly associated with increased odds of being anemic (OR = 1.85; CI = 1.16–2.95) comparing high quartile of AFB1-lys to low quartile
Groopman and others (2014)[9]	What is the distribution of aflatoxin exposure during pregnancy and what are the potential determinants? (longitudinal)	30 women with first and third trimester samples and 141 with first trimester samples (Nepal) 63 women with first, third, and cord blood samples (Bangladesh)	AFB1-lys	Nepal Maternal: 132 (94) Bangladesh Maternal and cord: 63 (100)	Median (range) Nepal Maternal: 22.45 pg/mg albumin (0.45–2,939.3) Bangladesh First trimester-18.08 (1.56–63.22) Third trimester-25.35 pg/mg albumin (3.37–72.8) Cord: 27.41 pg/mg albumin (4.62–76.69)	Children showed evidence of aflatoxin exposure during the first 1,000 days of life. There was an 2association between increased AGP and CRP levels and aflatoxin exposure

CI = confidence interval; IQR = interquartile range; OR = odds ratio; SD = standard deviation; AGP = apha-1-acid glycoprotein; CRP = C-reactive protein; HAZ = height-for-age z score.
*Biomeasures are aflatoxin (AF) B1, AFB2, AFM1, AFM2, AFG1, AFG2, and aflatoxicol in blood. Biomarkers are aflatoxin-lysine (AF-lysine) in blood and aflatoxin M1 (AFM1) in urine.

Supplemental Table 2

Animal studies investigating the effect of aflatoxin exposure during pregnancy

Author (year)	Animal model (*n*)	Aflatoxin dose and duration (treatment group)*	Results
Butler and others (1971)[10]	Rats (20)	0 (A), 5–7 mg/kg on day 16 (B), 5–7 mg/kg on day 19 (C)	Decrease in fetal weight in AF-treated animals. Authors suggest due to decrease in feed intake
Schmidt and others (1980)[11]	Hamster (40)	0 (A), 4 mg/kg body weight of crystalline AFM1 by IP injection on day 8 of pregnancy (B), 4 mg/kg on day 9 of pregnancy (C), 6 mg/kg on day 8 of pregnancy (D), 6 mg/kg on day 8 of pregnancy (E)	Decrease in fetal growth in all AF-treated groups by 0.5–0.7cm. Evidence of renal and hepatic necrosis in pregnant hamsters
Appelgren and Arora (1983)[12]	Mice (33)	0 (A), 4 mg/kg on day 8 (B), 4 mg/kg on day 9 (C)	Increase in fetal malformations and increase in pup mortality in AF-treated mice
Mocchegiani and others (1998)[13]	Sows and their piglets (24)	0 (A), 800 µg/kg AFG (B), 800 µg/kg AFB (C), 800 µg/kg AFG + 800 µg/kg AFB (D)	Decrease in piglet body weight in AFB-treated groups
Kihara and others (2000)[14]	Rats (30)	0 (A), 0.3 mg/kg/day AFB1 dissolved in dimethylsulfoxide subcutaneously on days 11–14 (B) or 15–18 of (C) gestation	Decrease in mean birthweights in offspring treated with AFB1; decrease in number of live births in group C
Oliveira and others (2002)[15]	Japanese quail (256)	0 (A), 25 µg/kg feed (B), 50 µg/kg feed (C), 100 µg/kg feed (D)	Decrease in egg weights in groups C and D
Wangikar and others (2004)	Rats (38)	0 (A), 0.125 mg/kg (B), 0.25 mg/kg (C), 0.5 mg/kg (D), 1 mg/kg (E)	Decrease in fetal weight and crown to rump lengths and increase in gross anomalies in group E compared with control. Cases of abortion in groups D and E
Wangikar and others (2005)	Rabbits (20)	0 (A), 0.025 mg/kg body weight (B), 0.05 mg/kg body weight (C), 0.1 mg/kg body weight (D)	Decrease in fetal weights in group D, decrease in crown to rump length in groups C and D, increase in skeletal anomalies, and impaired organ development in AF-treated fetuses
El-Nahla and others (2013)	Rabbits (6)	0 (A), 0.05 mg/kg from days 6–18 of pregnancy (B)	Decrease in fetal weights and crown rump length in AF-treated fetuses. Decrease in weight of fetal organs (liver and gall bladder, stomach and intestine, and lungs and kidneys). Increase in skeletal anomalies

AF = aflatoxin; IP = intraperitoneal.

*Letter in column labeled “Aflatoxin dose and duration (Treatment group)” refers to treatment group within the experiment.

Supplemental Table 3

Animal studies investigating the effect of aflatoxin exposure on hematological parameters

Author (year)	In vivo or in vitro animal model (*n*)	Aflatoxin dose and duration (treatment group)*	Results
Tung and others (1975)[16]	In vivo chicks (240)	0 (A), 0.625 μg/g feed (B), 1.25 μg/g feed (C), 2.5 μg/g feed (D), 5.0 μg/g feed (E), 10 μg/g feed (F)	Hemoglobin, erythrocyte count, and packed cell volume were significantly decreased in all AF-treated groups. Increase in leukocyte count in group F
Lanza and others (1981)[17]	In vivo chicks (*N* = 64)	0 (A), 5 μg/kg body weight/day (B)	Aflatoxin treatment reduced body weight and iron absorption regardless of presence of anemia. Effect on anemia was strain dependent
Verma and Raval (1991)[18]	In vitro rabbit red blood cells rabbit	0.35–3.5 μg/mL	Lower levels of AFB1 (< 1.4 μg/mL) caused morphological changes to RBC resulting in elimination; higher levels (> 1.4 μg/mL) caused hemolysis
Harvey and others (1995)[19]	In vivo pigs (*N* = 12)	0 (A), 2.5 mg/kg feed (B)	AF treatment reduced body weight and feed consumption and unsaturated iron-binding capacity, and increased total iron concentration indicating hemolytic anemia
Marin and others (2002)[20]	In vivo pigs (*N* = 36)	0 (A), 140 ppb (B), 280 ppb (C)	No effect on total red blood cell numbers or relative number of lymphocytes, monocytes, neutrophils, basophils, and eosinophils in AF-treated pigs. Biphasic effect on white blood cells with an increase in group C
Yousef and others (2003)[21]	In vivo rabbits (*N* = 15)	0 (A), 15 μg/kg body weight (B), 30 μg/kg body weight (C)	Decrease in hemoglobin in groups B and C. Decrease in erythrocytes and packed cell volume in group C
Eisa and Metwally (2011)[22]	In vivo rabbits (*N* = 20)	0 (A), 0.1 mg/kg feed (B)	Microcytic hypochromic anemia in AF-treated rabbits
Andretta and others (2012)[23]	In vivo broilers (meta-analysis 0of 98 studies containing 37,371 broilers)	Mean dose = 0.95 mg/kg feed (range = 0–5 mg/kg); mean duration = 18 days	Decrease in hematocrit, hemoglobin, leukocytes, heterophils, and lymphocytes in AF-treated broilers

AF = aflatoxin; RBC = red blood cell.

*Letter in column labeled "Aflatoxin dose and duration (Treatment group)" refers to treatment group within the experiment.

SUPPLEMENTAL REFERENCES

1. De Vries HR, Maxwell SM, Hendrickse RG, 1989. Foetal and neonatal exposure to aflatoxins. *Acta Paediatr Scand 78:* 373–378.
2. Maxwell SM, Familusi JB, Sodeinde O, Chan MCK, Hendrickse RG, 1994. Detection of naphthols and aflatoxins in Nigerian cord-blood. *Ann Trop Paediatr 14:* 3–5.
3. Abdulrazzaq YM, Osman N, Ibrahim A, 2002. Fetal exposure to aflatoxins in the United Arab Emirates. *Ann Trop Paediatr 22:* 3–9.
4. Abdulrazzaq YM, Osman N, Yousif ZM, Trad O, 2004. Morbidity in neonates of mothers who have ingested aflatoxins. *Ann Trop Paediatr 24:* 145–151.
5. Turner PC, Collinson AC, Cheung YB, Gong Y, Hall AJ, Prentice AM, Wild CP, 2007. Aflatoxin exposure in utero causes growth faltering in Gambian infants. *Int J Epidemiol 36:* 1119–1125.
6. Shuaib F, Jolly P, Ehiri J, Yatich N, Jiang Y, Funkhouser E, Person S, Wilson C, Ellis W, Wang J, Williams J, 2010. Association between birth outcomes and aflatoxin B1 biomarker blood levels in pregnant women in Kumasi, Ghana. *Trop Med Int Health 15:* 160–167.
7. Lamplugh SM, Hendrickse RG, Apeagyei F, Mwanmut DD, 1988. Aflatoxins in breast-milk, neonatal cord blood, and serum of pregnant-women. *BMJ 296:* 968.
8. Shuaib F, Jolly PE, Ehiri JE, Jiang Y, Ellis WO, Stiles JK, Yatich NJ, Funkhouser E, Person SD, Wilson C, Williams JH, 2010. Association between anemia and aflatoxin B1 biomarker levels among pregnant women in Kumasi, Ghana. *Am J Trop Med Hyg 83:* 1077–1083.
9. Groopman JD, Egner PA, Schulze KJ, Wu LS, Merrill R, Mehra S, Shamim AA, Ali H, Shaikh S, Gernand A, Khatry SK, LeClerq SC, Jr KP, Christian P, 2014. Aflatoxin exposure during the first 1000 days of life in rural south Asia assessed by aflatoxin B-lysine albumin biomarkers. *Food Chem Toxicol 74:* 184–189.
10. Butler WH, 1971. The effect of maternal liver injury and dietary reduction of foetal growth in the rat. *Food Cosmet Toxicol 9:* 57–63.
11. Schmidt RE, Panciera RJ, 1980. Effects of aflatoxin on pregnant hamsters and hamster fetuses. *J Comp Pathol 90:* 339–347.
12. Appelgren LE, Arora RG, 1983. Distribution studies of 14c-labelled aflatoxin B1 and ochratoxin A in pregnant mice. *Vet Res Commun 7:* 141–144.
13. Mocchegiani E, Corradi A, Santarelli L, Tibaldi A, DeAngelis E, Borghetti P, Bonomi A, Fabris N, Cabassi E, 1998. Zinc, thymic endocrine activity and mitogen responsiveness (pha) in piglets exposed to maternal aflatoxicosis B1 and G1. *Vet Immunol Immunopathol 62:* 245–260.
14. Kihara T, Matsuo T, Sakamoto M, Yasuda Y, Yamamoto Y, Tanimura T, 2000. Effects of prenatal aflatoxin B1 exposure on behaviors of rat offspring. *Toxicol Sci 53:* 392–399.
15. Oliveira CA, Rosmaninho JF, Butkeraitis P, Correa B, Reis TA, Guerra JL, Albuquerque R, Moro ME, 2002. Effect of low levels of dietary aflatoxin B1 on laying Japanese quail. *Poult Sci 81:* 976–980.
16. Tung HT, Cook FW, Wyatt RD, Hamilton PB, 1975. Anemia caused by aflatoxin. *Poult Sci 54:* 1962–1969.
17. Lanza G, Washburn KW, Wyatt RD, 1978. Relationship of iron-absorption to development of aflatoxin related anemia. *Poult Sci 57:* 1104.

18. Verma RJ, Raval PJ, 1991. Cytotoxicity of aflatoxin on red blood corpuscles. *Bull Environ Contam Toxicol 47:* 428–432.
19. Harvey RB, Edrington TS, Kubena LF, Elissalde MH, Rottinghaus GE, 1995. Influence of aflatoxin and fumonisin B1-containing culture material on growing barrows. *Am J Vet Res 56:* 1668–1672.
20. Marin DE, Taranu I, Bunaciu RP, Pascale F, Tudor DS, Avram N, Sarca M, Cureu I, Criste RD, Suta V, Oswald IP, 2002. Changes in performance, blood parameters, humoral and cellular immune responses in weanling piglets exposed to low doses of aflatoxin. *J Anim Sci 80:* 1250–1257.
21. Yousef MI, Salem MH, Kamel KI, Hassan GA, El-Nouty FD, 2003. Influence of ascorbic acid supplementation on the haematological and clinical biochemistry parameters of male rabbits exposed to aflatoxin B1. *J Environ Sci Health B 38:* 193–209.
22. Eisa AMA, Metwally AY, 2011. Effect of glucomannan on haematological, coagulation and biochemical parameters in male rabbits fed aflatoxin-contaminated ration. *World Mycotoxin J 4:* 183–188.
23. Andretta I, Kipper M, Lehnen CR, Lovatto PA, 2012. Meta-analysis of the relationship of mycotoxins with biochemical and hematological parameters in broilers. *Poult Sci 91:* 376–382.
